# Primary Nasal Tuberculosis

**DOI:** 10.4103/0970-2113.44127

**Published:** 2008

**Authors:** Ramakant Dixit, Lokendra Dave

**Affiliations:** 1 Assistant Professor, Department of Tuberculosis & Chest Diseases, J. L. N. Medical College, Ajmer, (India); 2 Assistant Professor, Respiratory Unit, Department of Medicine, Gandhi Medical College, Bhopal, (India)

Tuberculous involvement of nose, nasopharynx and para nasal sinus is extremely rare even in countries with a high incidence of pulmonary disease^1^. Nasal and sinus tuberculosis remains both silent and asymptomatic until well advanced. It develops, almost always, secondarily to a tuberculous focus elsewhere in the respiratory tract. Although nasal mucosa is inherently very resistant to tubercle bacillus, both trauma and atrophic changes facilitate successful lodging of bacilli within the nasal lining^2^.

Tuberculous involvement of nose is usually secondary to the tuberculosis of lungs or larynx, though in rare instances primary infection can occur at nose^3^,^4^. This communication describes such a case in a 10-year-old girl.

A 10-year-old female child presented with history of bleeding from nose off and on for last one year, mass in right nostril followed by disfigured nose for last three months. She had poor socioeconomic background, never gone to school and belongs to a migrating community.

Her general physical examination along with systemic examination was unremarkable. Local examination of nose revealed loss of columella and septal cartilage. A pinkish, exuberant, soft, sessile mass was present in the right nostril that was slightly bleeding to touch. There was no nasal discharge. Examination of oral cavity, larynx and ear was normal. There was no lymphadenopathy. A provisional diagnosis of granuloma nose was made.

Her investigations revealed hemoglobin 9.6 gm%, total leukocyte count 11500 cells/mm^3^ (polymorphs 61%, lymphocytes 28%, monocytes 2% and eosinophils 9%), ESR 41 mm in first hour with normal fasting blood sugar, renal function tests, bleeding profile and chest x-ray etc. She was VDRL and HIV seronegative. X-ray Para nasal sinus showed hypo-plastic frontal sinuses with haziness of both maxillary sinuses. Mantoux test revealed an induration of 20 mm with blister formation. Nasal endoscopy confirmed the findings of anterior rhinoscopy. The nasal turbinate and sinus openings were normal and there was no discharge.

Multiple biopsies were obtained from the nasal mass that on histological examination revealed granulomatous inflammation consisting of epitheloid cells, Langhans type giant cells and lymphocytes with areas of micro-caseation. The granulomas were confluent and other cellular components included plasma cells, foamy macrophages with intense neutrophillic infiltration and necrosis at several places. Sections were positive for acid- fast bacilli (AFB) on ZN staining. Tissue sections were negative for 10% KOH mount for fungus and fungal culture.

Patient was subsequently investigated thoroughly for any other focus of tuberculosis in the body but no such clue was found. She was put on 2HRZE/4HR regimen that she took regularly for three months before lost to follow up. Her parents also refused for reconstructive surgery.

**Fig 1:**
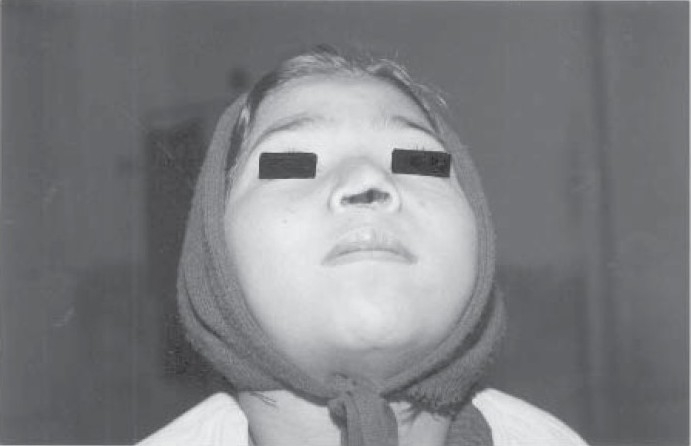
Photograph of the patient showing extensive local destruction of columella and nasal septum with nodular granulomas in nasal cavity.

The nose is least liable to invasion by acute tuberculosis of any part of the respiratory tract, because of the structure of mucosa, respiratory movements of the cilia and bactericidal secretions. However, nose can become infected either directly (primarily) through the air current by people sneezing or coughing or by direct inoculation by finger borne infections and by instrumentation. Indirectly (secondarily) the nose may become infected through the blood and lymph vessels^5^. It is twice as common in females as in males and common in persons living in unhygienic surroundings with poor health^6^.

Tuberculous involvement of nasal cavity usually appears as a rapidly growing ulcer or tumour mass in the quadrangular cartilage of the nasal septum. Frequently, a septal perforation develops but in contrast to lupus vulgaris, the adjoining skin is not affected. The anterior portions of the inferior turbinate are frequently involved. Involvement of posterior nares is rare and nasal floor is almost always spared^2^,^7^. Direct extension of infection from nose to ethmoid sinus may occur. The organism may spread into the sphenoid, frontal or maxillary sinuses through the sinus ducts. The orbit may be invaded and infection can extend to cranial cavity^3^,^8^. In our case the infection was localized but highly destructive causing facial disfiguration due to loss of columella and septal cartilage.

The clinical symptoms of nasal tuberculosis may not manifest themselves until the disease is well on its way. Bloody nasal discharge may be the earliest, possibly the only, presenting symptom. Pain, nasal obstruction and dryness in the nose or throat are other common presentation. The eye symptoms may occur due to blockage of the nasolacrimal duct or direct orbital invasion. Headaches may occur with invasion of the sinuses and extension to cranial cavity^5^,^7^.

Now a day nasal endoscopy easily facilitates accurate evaluation of these vague symptoms. This technique not only allows a more thorough examination of the nasal interior and nasopharynx but also facilitates biopsy of the mucosa or granulomatous mass^7^. The diagnosis of primary nasal tuberculosis in our case was made by demonstration of AFB and typical caseating granulomas in the biopsy tissue from nose along with negative workup for tuberculous foci elsewhere in the body. Although culture of nasal or nasopharyngeal secretions for AFB may yield positive results, biopsy is usually required to establish the diagnosis^9^. This is especially important because the differential diagnosis of disease may rest between tuberculosis and midline granulomas (i.e. wegners granulomatosis, leprosy, sarcoidosis, subcutaneous phycomycosis, granulomatous syphilis etc.) or carcinomas. Other differential diagnosis includes rhinoscleroma, rhino- sporidium seeberi, rhinitis sicca, suppurative sinus disease and foreign bodies etc^5^.

The management essentially consists of adequate anti-tuberculosis therapy with excisional surgery, if obstruction is significant and reconstruction plastic surgery for perforation of nasal septum and cosmetic purpose if required. It is very interesting to review the management of this condition in the pre chemotherapy era^5^ where cautery of nasopharyngeal or nasal ulceration was recommended for pain relief, coupled with a regimen of strict nasal hygiene. This included cleansing irrigations with alkaline antiseptic solutions, tampons of hydrogen peroxide to soften up the dried crusts, application of acids such as lactic, trichloracetic and chromic to the ulcers and use of galvanocautery and diathermy for destruction of the lesions. Other methods such as use of tuberculin, astringent powders (iodobor and aristol) insufflations in nose, pyrogallic ointment insertion in nose etc. along with sunlight, x-rays or ultraviolet rays, all have been used in past in an attempt to cure the disease or prevent extension of infection to the vital areas so as to reduce mortalities. Although these methods were symptomatically helpful, they have little application in modern management of the disease.
